# Precision measurement physics: physics that precision matters

**DOI:** 10.1093/nsr/nwaa271

**Published:** 2020-12-18

**Authors:** Mingsheng Zhan, Xincheng Xie

**Affiliations:** Innovation Academy for Precision Measurement Science and Technology, Chinese Academy of Sciences, China; School of Physics, Peking University, China

Without measurement, there would be no science. Modern science is established and developed in the process of hypothesis–test–model–theory cycle. Physics and measurement are inseparable. Precision measurement refers to the use of advanced technology and methods to pursue high accuracy under the existing physical framework. Precision measurement physics is to test the range of existing physics in a higher precision and try to find out the limit of the frame so as to discover new physics. In other words, precision measurement physics is the physics that precision matters. It is well known that an improvement of measuring precision in physics by an order of magnitude often leads to new physical discoveries. Early observations of the atomic separate line series gave birth to quantum mechanics, and the fine structure of atomic spectrum and the discovery of Lamb shift laid an experimental foundation for the birth of relativistic quantum mechanics and quantum electrodynamics (QED), respectively.

Physical measurement is to obtain the value on the agreed unit to determine the size of the physical quantity. Physical quantities are connected by physical constants to become physical laws. Therefore, the definition of the unit, the accuracy of the measured value, the value of the physical constant and whether the connection relation holds become the key to test the law of physics and the core content of the precision measurement physics study.

Unit is the basis of physical measurement, and international unit system (SI) is the basis of metrology and physical measurement comparison. A new revolution has recently taken place in the international system of units. Four of the SI basic units, kilogram, ampere, kelvin and mole, are redefined by the Planck constant *h*, the basic charge constant *e*, the Boltzmann constant *k* and the Avogadro constant *N*_A_, respectively. This is a new milestone in the history of metrology. In the interview made by Jin Wang, Tianchu Li first tells the story of the quantization and constant definition of the second and meter; he then explains the redefinitions of the four units and their significance for precision measurements.

Fundamental physical constants are some universal constants in physics. Among them are those related to the basic interaction in nature and the basic laws of physics; they are the gravitational constant *G*, the fine structure constant *α*, the Planck constant *h* and the speed of light *c*. Among these constants, precise determination of *G* is considered the most difficult. A new and so far the most accurate *G* has been given by Jun Luo and colleagues in 2018 using two independent methods. In their paper, they review the full history of the *G* measurement. Zhengtian Lu makes a wonderful comment on this work.

The law of physics is the relationship between physical quantities associated with fundamental physical constants. Few electron atomic and molecular systems provide an ideal platform for QED model and physical constant constraints test. Precise calculation can give the theoretical value of QED prediction, which requires the use of proton–electron mass ratio (*m*_p_/*m*_e_), fine structure constant (*α*), Rydberg constant (*R_∞_*), etc. Comparing the theoretical value with precise spectral measurement value, we can not only test the self-consistency between the QED theory and the constants, but also find out who is the ‘short board’ that affects the accuracy of the test (bucket effect). Recently, the group led by Shuiming Hu has measured the fine structure splitting of helium by laser spectroscopy and achieved a very high accuracy. Their results are in good agreement with the QED calculations up to order *m*_e_*α*^7^. Zongchao Yan comments on the work and prospects the future development.

Atomic frequency standard (also known as atomic clock) represents the forefront of measurement precision and accuracy. The development of precision measurement physics has always been led by the precision measurement of time and frequency. The improvement of atomic frequency standard accuracy leads to the improvement of other basic physical quantity definition, physical constant measurement and physical law test accuracy. In recent years, research in the field of time and frequency has developed rapidly. This special topic collects three representative works: one is the world's first cold-atom clock in space (Liang Liu *et al.*); the other is the first Chinese optical clock and the first calcium ion clock to enter 10^−18^ instability (Kelin Gao); another is on multiple-channel optical frequency divider based on an optical frequency comb, which is essential for high-precision frequency comparison (Longsheng Ma *et al.*).

All the above works, although based on Chinese teams, represent the international frontier of precision measurement physics. We thank the authors for their contributions to this special topic. It is a pity that due to time and space limits, some important developments, such as the equivalent principle test, atomic interferometer, neutral atom optic clock and so on, have not yet been included. We expect that these can be considered in the future.

